# Topic sensitivity still affects honest responding, even when specialized questioning techniques are used

**DOI:** 10.1111/csp2.12927

**Published:** 2023-03-31

**Authors:** Harriet Ibbett, Leejiah J. Dorward, Edward M. Kohi, Julia P. G. Jones, Stephen Sankeni, Joseph Kaduma, Jesca Mchomvu, Rose Mawenya, Freya A. V. St. John

**Affiliations:** 1 School of Natural Sciences, College of Environmental Science and Engineering, Bangor University, Bangor, UK; 2 Wildlife Population Monitoring, Tanzania Wildlife Research Institute (TAWIRI), Arusha Tanzania; 3 Conservation and Human Behaviour Research Group, Bangor University, Bangor, UK

**Keywords:** bias, hunting, non-compliance, Randomized Response Technique, rule-breaking, sensitive topics, social sciences, Tanzania

## Abstract

To develop more effective interventions, conservationists require robust information about the proportion of people who break conservation rules (such as those relating to protected species, or protected area legislation). Developed to obtain more accurate estimates of sensitive behaviors, including rule-breaking, specialized questioning techniques such as Randomized Response Techniques (RRTs) are increasingly applied in conservation, but with mixed evidence of their effectiveness. We use a forced-response RRT to estimate the prevalence of five rule-breaking behaviors in communities living around the Ruaha–Rungwa ecosystem in Tanzania. Prevalence estimates obtained for all behaviors were negative or did not differ significantly from zero, suggesting the RRT did not work as expected and that respondents felt inadequately protected. To investigate, we carried out a second study to explore how topic sensitivity influenced respondents' propensity to follow RRT instructions. Results from this experimental study revealed respondents understood instructions well (~88% of responses were correct) but that propensity to follow RRT instructions was significantly influenced by the behavior asked about, and the type of answer they were required to provide. Our two studies highlight that even if RRTs are well understood by respondents, where topics are sensitive and respondents are wary of researchers, their use does not necessarily encourage more honest responding.

## 1 Introduction

Rules are essential for the sustainable management of natural resources, threatened species and protected areas (Keane et al., 2008; Ostrom et al., 1999). To develop more effective interventions, conservation practitioners and policy-makers require an understanding of what motivates people both to comply with, and to break, rules (Arias, 2015; St John et al., 2013). However, obtaining information from people about compliance can be challenging. People may perceive discussions concerning prohibited resource extraction or use to be sensitive (Lee & Renzetti, 1990) and as a result, a non-random proportion of respondents may refuse to participate or answer specific questions, introducing non-response bias (Blair et al., 2020; Fisher, 1993; Tourangeau et al., 2010). Further, respondents may not provide accurate or honest answers because they are scared of being punished for declaring their involvement in rule-breaking (sensitivity bias, [Blair et al., 2020]); or because of a desire to project a more favorable image of themselves to others, or to conform to prevailing social norms (social desirability bias, [Krumpal, 2013]).

To overcome this, conservation researchers are increasingly using specialized questioning techniques; a suite of tools developed by social scientists to reduce bias and obtain more accurate estimates of the prevalence of sensitive behaviors such as rule-breaking (Cerri et al., 2021; Hinsley et al., 2018; Ibbett et al., 2021; Nuno & St John, 2015). With their flexible designs, which make it possible to capture different types of information, Randomized Response Techniques (RRTs) are the most common specialized questioning technique applied to date in conservation (Ibbett et al., 2021). RRTs typically rely on a randomization process (e.g., the flipping of a coin, or rolling of a dice, see Box 1), to determine the type of answer a respondent should give (e.g., a prescribed, or truthful response) (Fox, 2017). Crucially, the result of the randomizing process is never revealed to the researcher, but by using the known probability of each option being selected, researchers can estimate the prevalence of the sensitive trait (Warner, 1965). By introducing an element of uncertainty into the response process, RRTs are proposed to provide respondents a greater sense of protection above and beyond simple guarantees of anonymity (St John et al., 2011), and thereby encourage more honest answers (Fox, 2017). Consequently RRTs have been used to explore a variety of conservation topics including illegal consumption of giraffe meat in Kenya (Ruppert et al., 2020) and bear bile in Cambodia (Davis et al., 2019), non-compliance with fishing regulations in New Zealand and Chile (Oyanedel et al., 2017; Thomas et al., 2015), and hunting of protected species in Indonesia (St John et al., 2018) and China (Chang et al., 2019).

Despite their widespread use, the ability of RRTs to reduce bias and increase response accuracy when discussing sensitive topics is unclear (Ibbett et al., 2021; Lensvelt-Mulders, Hox, van der Heijden, & Maas, 2005; Umesh & Peterson, 1991). While some comparative studies suggest RRTs produce higher, and presumably more accurate estimates than conventional methods such as direct questions (e.g., Carvalho, 2019; Cerri et al., 2017), evidence from the few validation studies that exist, suggest RRTs often underestimate prevalence (Bova et al., 2018; Lensvelt-Mulders, Hox, van der Heijden, & Maas, 2005; Rosenfeld et al., 2016). Some researchers suggest that the method confuses respondents (Razafimanahaka et al., 2012), and uncertainty remains as to how instruction comprehension and topic sensitivity influence respondents' propensity to answer accurately, potentially introducing other forms of error. For example, respondents may provide inaccurate answers because they do not understand RRT instructions, or because they choose not to follow instructions (known as evasive response bias) (Clark & Desharnais, 1998; John et al., 2018). Deliberate inaccurate, or evasive answering has repeatedly been raised as a concern of RRTs, particularly forced-response designs (as described in Box 1) which by their very design instruct a known proportion of respondents to report possession of the sensitive characteristic, irrespective of whether this answer accurately reflects their own behavior; understandably, respondents can be reticent to follow such instructions (Clark & Desharnais, 1998; Feth et al., 2017; John et al., 2018). In recent years, advanced statistical measures have been developed to calculate the proportion of a sample who do not follow RRT instructions (i.e., the Cheater Detection Model, [Clark & Desharnais, 1998]). However, these measures require specific design choices, large sample sizes, and are typically conducted post-hoc. Yet researchers need to know before conducting their research whether the questioning approach they choose is likely to be successful.

Given the increasing application of RRTs in conservation, understanding their effectiveness is of high importance for researchers, as well as practitioners and policymakers who require reliable data to make informed decisions. Recently, a study empirically tested understanding of forced-response RRT instructions (Ibbett et al., 2022). Respondents living around protected areas in Indonesia and Tanzania were asked to imagine they were a fictional character, and to provide the response characters should give when asked whether they conducted an illegal behavior, hunting wildlife. Because the hunting status of each character was known, researchers could assess whether respondents provided accurate responses. Correct answers to the RRT questions were reasonably high (81% in Indonesia, and 80% in Tanzania). However, authors were unable to distinguish whether incorrect answers occurred because of poor understanding of RRT instructions, or because respondents understood instructions, but purposefully chose to ignore them due to concerns associated with the sensitivity of the research topic. This was particularly pertinent in Tanzania where additional research highlighted that discussing hunting wildlife was particularly sensitive in surveyed communities (Ibbett et al., n.d.).

Here, we aimed to assess the performance of a forced-response RRT when asking communities living around protected areas questions about conservation non-compliance. To do so, we developed two studies. The first used a forced-response RRT to measure the prevalence of five prohibited behaviors. In the second study, we explored how topic sensitivity influenced respondents' propensity to follow RRT instructions. We compare findings from both studies and highlight some of the complexities that researchers must consider when deploying forced-response RRTs to investigate rule-breaking.

## 2 Study Area

**Figure 1:**
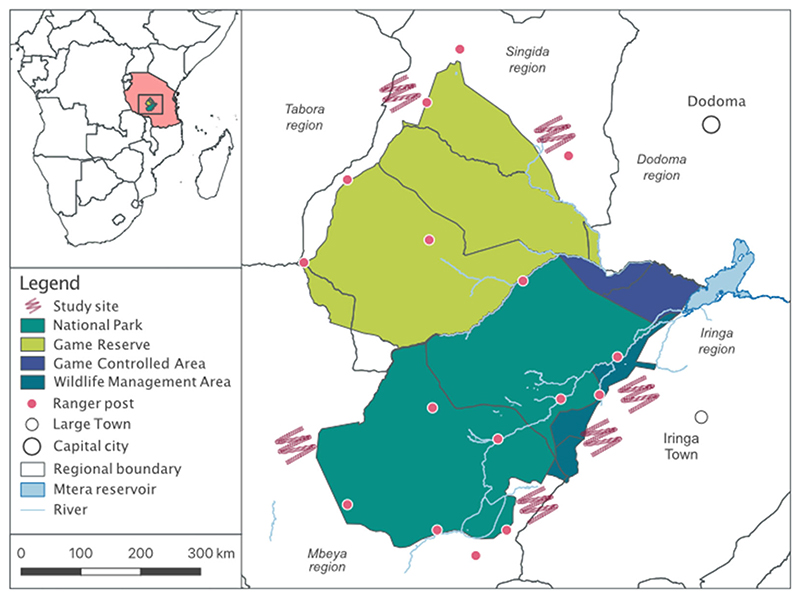
Data was collected in six villages situated around the Ruaha-Rungwa ecosystem in central southern Tanzania. In accordance with ethics approval, we do not indicate the precise locations of study villages.

Our research was conducted in communities surrounding the Ruaha-Rungwa ecosystem in central southern Tanzania (Figure 1). Covering ~45,000 km^2^, the landscape supports some of the largest remaining carnivore populations in Africa (Dickman et al., 2014), and is comprised of several protected area types, including community-managed Wildlife Management Areas, game controlled areas, privately leased hunting concessions (e.g., Rungwa, Kizigo and Muhesi Game Reserves), and Ruaha National Park. In 2008, Usangu Game Reserve was incorporated into Ruaha National Park, making it one of the largest protected areas in Tanzania (Zia et al., 2011). Communities living within the landscape are ethnically diverse, and include traditional pastoralists (e.g., Sangu, Masai and Barabaig), and agro-pastoralists (e.g., Hehe, Bena, Gogo) (Dickman et al., 2014; Walsh, 2007). Historically, hunting and collection of natural resources, such as honey, were important livelihood activities (Walsh, 2007). Today however, strict rules regulate the use of natural resources within protected areas, with the entrance to any National Park or Game Reserve for any reason (including collection of natural resources or grazing of livestock) prohibited without permission (Wildlife Conservation Act, 2009; National Parks Act, 2003). In addition, in Tanzania, all wildlife belongs to the state (Article 4, Wildlife Conservation Act, No. 5 2009), meaning it is illegal to hunt, kill or wound any wild animal anywhere, without permission (Article 55.1).

Non-compliance with protected area and wildlife rules has been identified as a conservation concern in the ecosystem (e.g., Beale et al., 2018; Hariohay et al., 2019). Previous studies have surveyed community members arrested whilst rule-breaking in Game Reserves (Hariohay et al., 2019), and interviewed key informants to explore whether poverty drives poaching (Knapp et al., 2017). Findings revealed arrests were made for a variety of transgressions including logging timber, hunting wildlife, grazing livestock, mining, and elephant poaching (Hariohay et al., 2019); and indicated high economic heterogeneity among households that illegally hunt (Knapp et al., 2017). To our knowledge, this is the first application of RRT to estimate prevalence of rulebreaking in the Ruaha-Rungwa ecosystem.

## 3 Methods

### 3.1 Assessing the prevalence of respondents’ rule-breaking behaviors (hereafter main study)

Previous research identified that people living in the Ruaha-Rungwa ecosystem enter protected areas for many reasons, but are often unwilling to discuss doing so due to concerns about possible repercussions from law enforcement (Ibbett et al., n.d.). To encourage more accurate responding, we used a forced-response RRT to ask individuals whether they conducted each of five prohibited behaviors in nearby protected areas. We selected this design over other variations, as it is commonly deployed in conservation studies (Ibbett et al., 2021), and piloting and previous research in the landscape (Ibbett et al., 2022) suggested it would be well understood and easy to implement. The prohibited behaviors, identified as prevalent in protected areas during focus group discussions (Ibbett et al., n.d.) were; entering the nearest protected area for any reason without permission, as well as entering the nearest protected area for specific reasons including to: graze livestock; collect building materials; go fishing; and hunt wildlife. Because these activities were reported to be gendered, with men more likely to do them and women more likely to play a supporting role (unpublished data), we tailored questions according to respondent gender. Men were asked if they personally conducted the behavior whilst for all behaviors except entering protected areas, women were asked if they encouraged the behavior in other members of their household.

We first collected basic demographic data, including respondents' age, gender, and years of schooling. To answer RRT questions on rule-breaking, respondents were provided a six-sided die in an opaque cup and asked to shake it prior to each question, without revealing the dice score to the interviewer. If a 1, 2, 3, or 4 was rolled (a probability of 0.66), respondents were instructed to answer truthfully whether they conducted (men only) or encouraged the behavior (women only, except for entering protected areas). If a 5 was rolled, respondents were instructed to answer “yes”, regardless of whether this was their “true” answer and if a 6 was scored, respondents were instructed to answer “no” (probability 0.167 each). Piloting revealed that while some respondents associated dice (commonly used as randomizers) in conservation RRT studies (Ibbett et al., 2021) with gambling, overall, respondents were familiar and happy to use one.

### 3.2 Experimental study assessing effect of topic sensitivity on response accuracy (hereafter experimental study)

**Figure 2:**
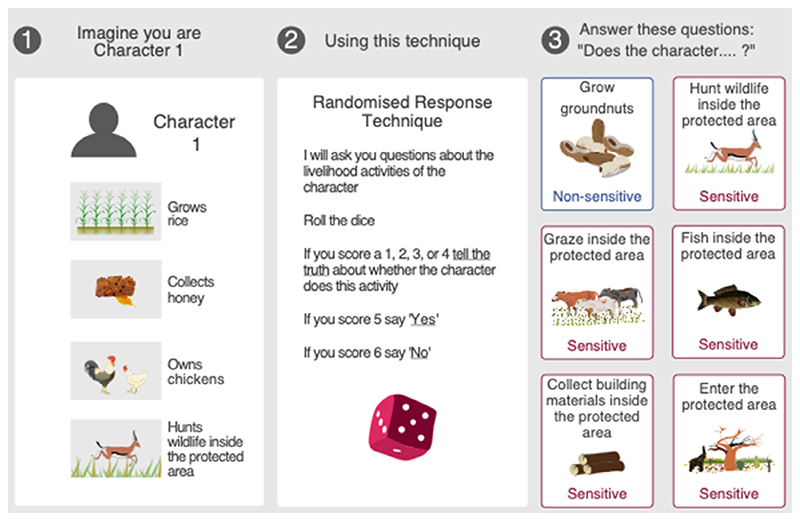
Example of a fictional character card (step 1, left), the instructions on how to answer the Randomized Response Technique (step 2, middle), and the six behaviors of interest respondents were asked about (step 3, right). One behavior was a non-sensitive legal behavior (outlined in blue), and five were potentially sensitive rule-breaking behaviors (outlined in red). Respondents were asked about the behavior of two characters, for each of the six behaviors. Presumed sensitivity was not indicated to respondents. It was explained to respondents that if a behavior was not listed on the character card, then the character did not conduct the behavior.

To assess whether respondents understood the RRT instructions and to explore how response accuracy varied with topic sensitivity, we adapted the experimental design of Ibbett et al. (2022). We first explained how the RRT protects participants and then introduced them to cards depicting the behaviors of fictional characters. Respondents were asked to imagine they were each fictional character, and following the RRT instructions, to answer questions about whether the fictional character conducted a specific behavior (Figure 2). In the experiment, we used the same forced-response RRT design as the main study described above and asked about the same five rule-breaking behaviors. However, to assess whether topic sensitivity influenced responding, we included an additional non-sensitive behavior, growing groundnuts. Farmed widely across the landscape, both for subsistence and commercial purposes (unpublished data), we assumed that individuals would have few concerns reporting whether fictional characters grew groundnuts. Thus, we hypothesized that any incorrect responses that occurred for this non-sensitive behavior were likely to be because respondents did not understand the instructions, rather than because they understood instructions but chose to disregard them due to sensitivity concerns.

In total, we presented each respondent with 14 character cards (Figure 2, Supplementary materials S1). Of the 14 fictional characters, the same two were always delivered first to introduce and practice the RRT. We only proceeded beyond these practice character cards once respondents answered correctly. Of the remaining 12 characters, two were allocated to each of the six behaviors of interest; one character always conducted the behavior of interest; one did not. To minimize order effects, we randomized the order characters were presented to respondents.

To assess whether individuals provided the correct answer, we asked them to verbally report their dice score, after providing their answer. While this usually undermines the anonymity assured by the method and should never be done when using RRT to collect data on respondents’ possession of sensitive traits, it was acceptable in this experimental study because we were collecting data on the behavior of fictional characters. At the end of the experiment, respondents were asked, using Likert scales to rate how well they understood the RRT method, how easy RRT questions were to answer, how much privacy they perceived RRT provided, and how comfortable they would feel providing honest responses about their own behaviors using RRT. Any specific feedback given to interviewers on the method or study was recorded. Interviewers also evaluated how well they thought the respondent understood the method, and whether they suspected the respondent of deliberately disregarding instructions. Basic demographic data (respondent age, gender, years of education) were also gathered, alongside respondent's familiarity with dice (using a yes/no response).

### 3.3 Data collection

Data were collected between January 2020 and November 2021. To estimate the overall prevalence of rule-breaking (i.e., the main study) we selected villages (n = 3) proportionate to population size and recruited respondents using a random sampling strategy (individuals were randomly selected from lists of village residents). To maximize admissions of rule-breaking, we biased survey effort towards men aged 18–55, who were more likely to be involved in rule-breaking (Hariohay et al., 2019; unpublished data, see Supplementary Materials S1). To reduce priming effects (e.g., respondents briefing other respondents about the survey content) and to minimize the potential for concerns to arise (e.g., if those who were asked about their own behaviors realized others were asked only about the behavior of fictional characters), our experimental study was conducted in neighboring villages (*n* = 8). Here respondents were recruited via convenience sampling, which was conducted with the help of a representative chosen by the village leader. Because we wished to explore how comprehension changed with gender, in the experimental study we sampled an equal proportion of men and women. Both survey instruments were developed in English and translated into Kiswahili by two team members, and then independently back-translated and piloted. Questionnaires were administered face-to-face by Stephen Sankeni, Jesca Mchomvu, Joseph Kaduma, Rose Mawenya, who were all Tanzanian nationals non-local to the study area. All data were collected using Open Data Kit (Brunette et al., 2013) on encrypted mobile phones (see Supplementary Materials S1).

### 3.4 Ethical considerations

All respondents were over 18 years old; with free, prior and informed consent obtained verbally. All data collected during the experimental study were anonymous, however, respondents in the main survey were given the option of providing contact details for a follow up survey if they wished. All identifiable data were encrypted at point of collection and pseudoanonymized for analysis. As a token of thanks, respondents were given a voucher for a cell phone provider of their choice. Research was approved by Bangor CoESE Ethics Committee and fieldwork complied with all Tanzanian COVID-19 regulations, with health and safety measures implemented to mitigate against transmission in survey communities.

### 3.5 Analysis

We performed all analyses in R v3.6.2 (R Core Team, 2019). In the main study, RRT prevalence estimates for each behavior were calculated following Hox and Lensvelt- Mulders (2004):

$$\pi =\frac{\lambda -\theta }{s}$$

where *π* is the estimated prevalence of the behavior in the sample, *λ* is the proportion of all “yes” responses in the sample, *θ* is the probability of providing a “forcedyes” response (0.167), and *s* is the probability of answering the sensitive question truthfully (0.66). Bootstrapping, with 10,000 samples was used to calculate 95% confidence intervals.

For the experimental study, we calculated the overall proportion of correct responses for each behavior. Using descriptive statistics we explored data, assessed respondent's understanding of RRT including compliance with instructions, and tested for collinearity between predictors prior to modeling. To examine what affected whether a respondent answered a question correctly, we fitted generalized linear mixed models using lme4 (Bates et al., 2015). The response variable was a binary indicator of whether a respondent gave a correct or incorrect answer to each question. Respondent gender, age, years of education and the type of response required (i.e., a yes to the sensitive behavior, a yes to the non-sensitive behavior, a no to the sensitive behavior, or a no to the non-sensitive behavior) were all included as fixed effects. We included a random effect to control for individual. Models were fitted using a BOBYAQA optimizer to achieve convergence, were tested for singularity and showed no significant signs of dispersion when checked using DHARMa (Harting, 2020).

## 4 Results

### 4.1 Main study

#### 4.1.1 Prevalence of respondents’ rulebreaking behavior

We asked 319 men and 105 women about their rule-breaking behavior. Respondents were aged between 18 and 80 (men: mean 33 [SD 8.4], women: 30 [SD 7.5]), and had a mean of 7 years of education (men: 6.5 [SD 3.1], women: 6.7 [SD 2.8]). Non-response rates were relatively high (~10%) with 32 men and 10 women refusing to answer questions about any behavior. Additionally, two women refused questions about entering protected areas, while one further woman did not answer about encouraging household members to collect building materials from inside protected areas.

**Figure 3:**
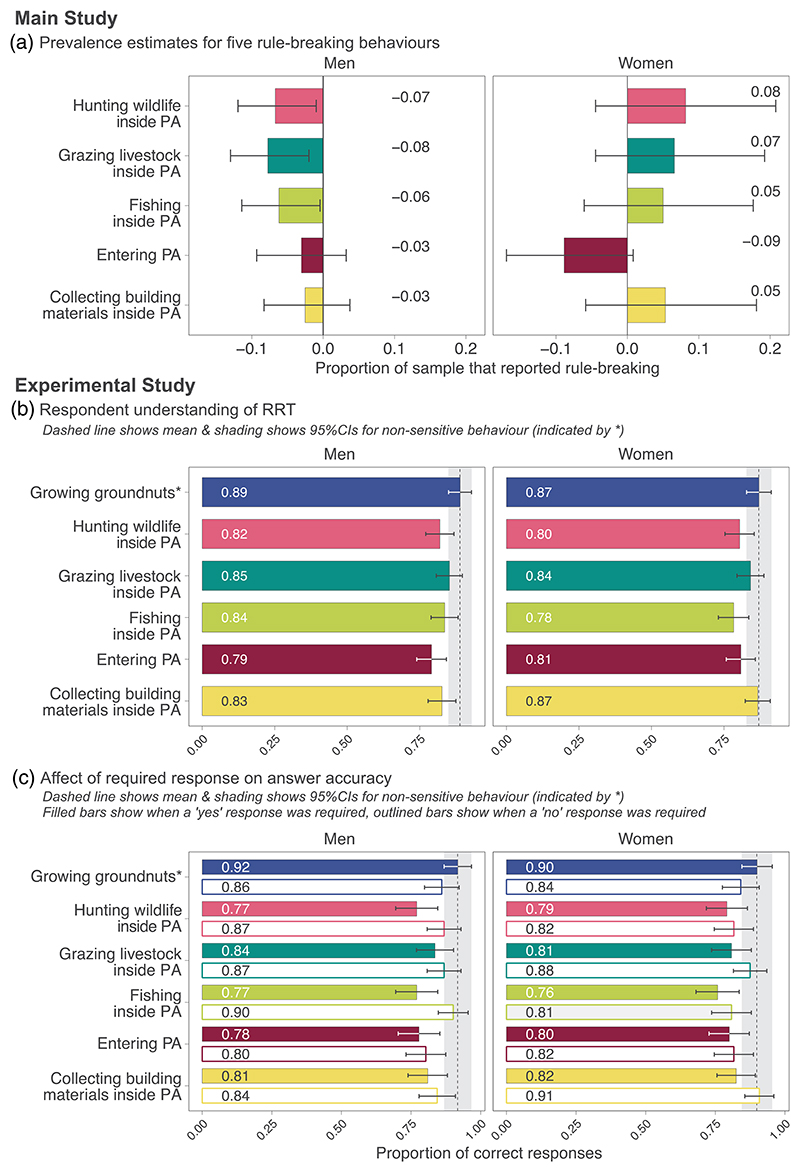
(a)) Prevalence of rule-breaking behaviors obtained in the main study using Randomized Response Technique (RRT) (men, *n =* 287; women, *n =* 95 [n = 94 for collecting materials, *n =* 93 for entering PA]) with 95% CIs. (b) Mean proportion of correct responses when using RRT to answer questions about behavior of fictional characters in the experimental study (men, *n =* 123; women, *n =* 120). (c) Proportion of correct responses for each behavior, separated by the type of answer required.

Overall, prevalence estimates for all rule-breaking behaviors were very low (Figure 3a). There was a notable difference in estimates between gender, with negative estimates obtained for men for three behaviors: hunting wildlife (–0.07 [lower 95% confidence interval: -0.12, upper confidence interval: -0.01]); grazing livestock (–0.08 [–0.13, -0.02]), and fishing (–0.06 [–0.11, -0.00]) and estimates that did not differ significantly from zero for two behaviors: entering protected areas (–0.03 [–0.09, 0.03]), and collecting building materials inside protected areas (–0.03 [–0.08, 0.04]). In contrast, prevalence estimates for all behaviors except entering protected areas for any reason without permission were positive for women (Figure 3a). However, because of the small sample size, large confidence intervals overlapped with zero, indicating that prevalence did not differ significantly from zero for any behavior.

### 4.2 Experimental study

#### 4.2.1 Proportion of correct responses for each behavior

We surveyed 123 men and 120 women during the experimental study to assess how well our forced-response RRT design was understood, only one respondent refused to answer one question. Respondents were aged between 18 and 45 (men: mean 31 [SD 8.3], women: 29 [SD 7.5]), and had a mean of 7 years of education (men: 7.4 [SD 2.6], women: 7.1 [SD 3]). Overall, respondents were more likely (but not significantly so) to answer correctly when asked about the non-sensitive behavior (growing groundnuts) (Figure 3b). The proportion of correct responses was slightly higher for men (0.89 [0.85, 0.93]) than women (0.87 [0.83, 0.91]).

#### 4.2.2 The type of response required significantly affected whether a respondent answered correctly

Modeling showed that when respondents had to answer “yes” about the characters’ behavior (regardless of whether this was a truthful “yes”, or a “forced” yes) for any of the rule-breaking behaviors, the likelihood of a respondent answering correctly was lower than when respondents were required to answer “no” about the sensitive behavior (either truthfully or “forced”) (Table 1, Figure 3c). When required to answer “yes”, about whether the character grew groundnuts, the opposite was true, with respondents more likely to answer correctly than when they were required to answer “no”. The type of “no” (i.e., whether it was no to a sensitive behavior, or no to a non-sensitive behavior) had no effect on response accuracy. Demographic characteristics such as age, gender and education had no effect on response accuracy. The proportion of total variance explained by both fixed and random effects in the model was low (conditional *R*^2^ = 0.282) suggesting other, unknown factors may also contribute towards variance.

**Figure 4:**
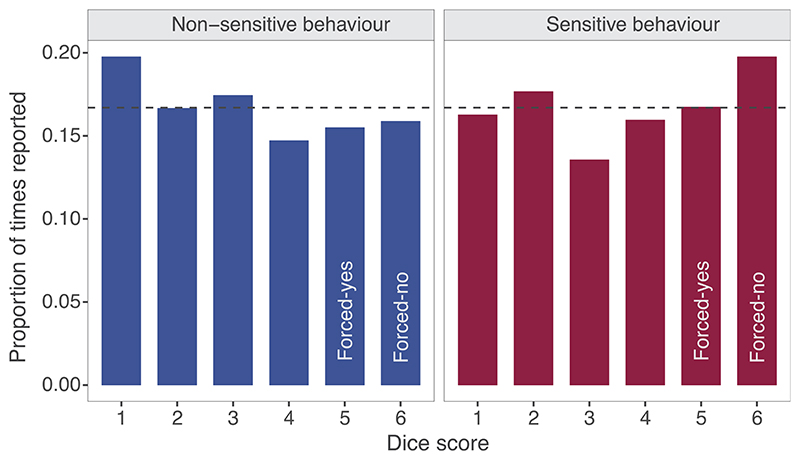
Proportion of times each number on the die was reported as rolled when respondents were answering questions about a non-sensitive behavior (growing groundnuts) versus a sensitive behavior (all other behaviors). Dashed line indicates the expected proportion of times each dice number should have been reported (0.167).

When answering questions about the non-sensitive behavior (growing groundnuts), dice scores reported by respondents did not differ from expected (*χ*^2^=2.4651, df = 5, p-value = .782), suggesting that respondents followed instructions (Figure 4). However, when asked to answer questions about a sensitive rule-breaking behavior, the dice scores reported differed significantly from expected (*χ*^2^=16.167, df = 5, p-value = .006), with more individuals reporting that they obtained a forced-no score (i.e., that their die landed on 6), and fewer individuals reporting scores that required truthful answers (i.e., die landing on 1, 2, 3, or 4). The number of forced-yes responses reported (i.e., die landing on 5) was as expected.

#### 4.2.3 Do interviewers accurately assess respondents understanding of, and compliance with, RRT instructions?

In the experimental study, interviewers reported that they thought respondents clearly understood or understood RRT in 70% of surveys. When interviewers' assessments were compared against a respondents' performance (measured as the proportion of correct responses across all behaviors) we found no significant association (*F*-value = 1.284, *p =* .281), suggesting interviewers did not accurately assess respondents’ understanding (Figure S1a). Interestingly, interviewers suspected 12% of respondents of deliberately not following instructions. When compared against respondents' actual performance, we found a significant association between interviewers' suspicions and the likelihood of answering correctly, with those suspected of not following instructions significantly less likely to answer correctly, compared to those that interviewers believed followed instructions (Figure S1a, *F*-value = 5.192, *p* = .006).

#### 4.2.4 Respondents’ perspectives on RRT

Most respondents in the experimental study reported they found RRT easy or very easy to understand (72%), and that they understood how to answer questions (90%), with most (72%) reporting that they would be comfortable answering sensitive questions about their own behavior honestly using RRT. Fewer (59%) respondents felt RRT kept their response secret (Table 2).

Nearly a fifth (18%) of respondents provided additional feedback. A third (31%) of comments were positive. Respondents reported that “it [RRT] is simple and easy to understand” and “it [RRT] is a good technique” (see Supplementary Materials S1). A third (30%) of comments highlighted concerns about the method. For example, three respondents were concerned the RRT was related to magic or witchcraft, while eight individuals reported concerns about being forced to provide answers that incorrectly suggested they might do the behavior. One respondent was concerned that their farm would be incorporated into the protected area as a result of the study, and stated that they deliberately answered incorrectly, whilst another said, “it is hard to answer “yes” to rule-breaking because the study may bring eviction”. Other comments related to level of education. For example, one respondent said, “it is difficult for us who did not go to school”. Another said, “it is difficult because it contains many things that are confusing”. Interestingly, one respondent stated, “people are now better educated so you should [just] ask them directly”.

## 5 Discussion

We estimated that the prevalence of all rule-breaking behaviors assessed in the main study were negative or did not differ significantly from zero; suggesting that the RRT did not work as expected. Other conservation studies have reported similar findings, for example, when asking about hunting of tiger, sambar, and pangolin in Indonesia, St. John et al. (2018) obtained negative prevalence estimates, and Davis et al. (2019) obtained estimates that did not differ from zero when estimating bear bile consumption in Cambodia. Moreover, a recent review assessing the application of RRTs across a range of disciplines reported mixed evidence regarding their success (Ibbett et al., 2021). While the likelihood of obtaining estimates that do not differ significantly from zero can be decreased by obtaining larger sample sizes (thereby reducing noise introduced during randomization and resulting in tighter confidence intervals) (Fox, 2017; Lensvelt-Mulders, Hox, & van der Heijden, 2005), obtaining negative estimates highlights more fundamental issues with how the method has been received by respondents. According to the forced-response RRT design, negative estimates can only occur when fewer than expected forced-yes responses are obtained, perhaps because respondents misunderstand instructions, or distrust that anonymity is ensured (Feth et al., 2017).

While poor comprehension of RRT instructions is often cited as a driver of non-significant or negative RRT estimates, particularly in low literacy contexts (e.g., Davis et al., 2019; Razafimanahaka et al., 2012), our experimental study suggests that low understanding was unlikely to be the only driver of the negative prevalence estimates derived in the main study. More than two thirds of respondents reported that the method was easy and understandable, and respondents generally answered correctly for the non-sensitive behavior. The forced-response RRT design assumes that when respondents feel adequately protected, they are equally as happy to provide a “yes” or “no” answer, regardless of whether answers are “forced” or truthful (Fox, 2017). We found compelling evidence that this was not the case in our experimental study as the type of response required significantly impacted the likelihood of a respondent answering correctly. Respondents were significantly less likely to answer correctly when they had to provide an affirmative answer about a character’s rule-breaking behavior. Both the negative prevalence estimates obtained in the main study and the failure to provide the correct responses about the sensitive behaviors of the character in the experimental study highlight respondents’ concerns about the potential consequences of providing affirmative responses to researchers. The higher than expected dice scores reported for the forced-no response (a dice score of 6) suggests some respondents answered “no” to avoid even the possibility of anyone associating them with rule-breaking, a trend suspected to occur if participants perceive a topic as especially sensitive (Clark & Desharnais, 1998; John et al., 2018).

Where respondent’s face potentially moderate to severe costs (whether psychological, social, monetary, or physical), they are more likely to be concerned about providing truthful answers (Tourangeau & Yan, 2007). Previous research has shown that discussing violations of protected area rules is sensitive in the study landscape, both because individuals are concerned about incurring sanctions (Ibbett et al. n.d.), but also because of poor relations between some communities and protected area authorities (Zia et al., 2011). Elsewhere in Tanzania it has been reported that communities with poor relationships with protected areas can view conservation research efforts as an attempt to appropriate resources (Brockington et al., 2008; Weldemichel, 2020); despite the protection RRT offers to respondents, it failed to overcome these multiple and related challenges associated with estimating rule-breaking prevalence.

Indeed, some respondents in the experimental study highlighted concerns that their responses about character's behavior may be used to trick them into revealing their own actions, while others raised concerns about being evicted from their lands as a result of research. Willingness to answer questions about sensitive topics is influenced by an individual's beliefs about whether their responses, and/or participation, will be revealed to third parties (Tourangeau & Yan, 2007). Thus, questions about sensitive topics such as rule-breaking often raise issues of trust (Krumpal & Voss, 2020), influenced by respondents' beliefs about who the researcher is, who they work for, who can access data, as well as what the researcher represents to the participant (Blair et al., 2020; Tourangeau & Yan, 2007). Communities living around conservation areas often perceive researchers to represent the interests of government, and conservation NGOs, regardless of whether they actually do (Brittain et al., 2020; Kiik, 2018), which can significantly affect respondents' trust in the research process. Our findings also reinforce ethical concerns about the appropriateness of using RRT designs that force respondents to provide responses that could be construed as admissions of incriminating behavior (Ibbett et al., 2022), particularly in contexts where distrust of researchers may already be high. Alternative RRT designs, such as the Unrelated-Question which use randomizers to determine the question answered, rather than force specific types of response, may assure respondents a greater sense of protection. Beyond RRT, other specialized questioning techniques, such as the Unmatched Count Technique, which requires respondents to report the number of items from a list that apply to them (Hinsley et al., 2018), have been shown to be well understood (Ibbett et al., 2022) and may be more appropriate. Researchers should also consider ways in which they can triangulate findings from quantitative surveys. Conducting in-depth interviews, or group exercises with key informants, for example, may provide additional data to help researchers better understand the context in which research is being conducted, and any sensitivities associated with discussing rule-breaking (Ibbett et al., n.d.).

Both studies had limitations. Previous studies have found that using randomizers such as dice can be problematic for some respondents due to associations with divination (Razafimanahaka et al., 2012). Although piloting suggested dice were appropriate, a small minority of respondents raised concerns in both studies, suggesting a different choice of randomizer may be more appropriate in future. A key limitation of the experiment was its complexity. Asking respondents about the behavior of multiple fictional characters undoubtedly added cognitive load to an already complex task. This may have deflated the proportion of people who answered correctly (because answering about a character was more difficult).

## 6 Conclusions

Specialized questioning techniques, such as RRT, are often promoted in conservation science as a way of improving the reliability of data collected from people about potentially sensitive topics. However, they do not always work as expected. Overall, while participants living around protected areas in central southern Tanzania understood the forced-response RRT method, their level of trust in the researchers and research process was insufficient for them to report true behavior. Ultimately, the challenges of using RRT go beyond respondents' understanding of the method and can be heavily influenced by their wider trust in the research process. Careful consideration of these factors is needed before methods are selected.

## Supplementary Material

Supporting Information

## Figures and Tables

**Table 1:** Log-odds regression coefficients with 95% confidence intervals from a binomial general linear mixed model, with random effects for respondent. The binomial represents whether the respondent answered the question correctly, or not, regardless of the behavior asked about.

Predictors	Log-odds	CIs 95%	*P*
(Intercept)	2.10	1.79-2.40	**<.001**
Age	0.10	–0.09 - 0.29	.303
Years of education	–0.06	–0.24 - 0.13	.554
Female^a^	–	–	–
Male	0.06	–0.30 to 0.43	.746
Required to answer “no” to sensitive behavior^a^	–	–	–
Required to answer “no” to non-sensitive behavior	–0.00	–0.41 to 0.41	1.000
Required to answer “yes” to non-sensitive behavior	0.62	0.13-1.11	**.013**
Required to answer “yes” to sensitive behavior	–0.45	–0.68 to –0.22	**<.001**
**Random effects**			
*σ*^2^			3.29
τ_00id_			1.18
ICC			0.26
*N_id_*			242
Observations			2903
Marginal R^2^/conditional R^2^			0.25/0.282

*Note:* Text in bold represent *p*-values which had statistical significance of <.05. ^a^Reference categories.

**Table 2:** Percentage of respondents in the experimental study (n = 243) that reported different perspectives regarding the Randomized Response Technique.

**How easy did you find it to answer the question using this method?**					
Don’t know	Very difficult	Difficult	OK	Easy	Very easy
–	3%	11%	14%	35%	37%
**How comfortable would you feel answering questions honestly about sensitive topics using this method?**					
Don’t know	Very uncomfortable	Uncomfortable	Neutral	Comfortable	Very comfortable
–	4%	5%	14%	50%	22%
**Do you feel you clearly understood how to answer the questions?**					
Don’t know	Did not understand	Difficult to understand	Under-stood		Understood well	
–	2%	8%	60%		30%	
**How secret do you think your answers were using this method?**						
Don’t know	Not at all secret	Neutral	Secret		Very secret	
12%	20%	9%	44%		15%	

## Data Availability

All data are available open access: doi: https://doi.org/10.6084/m9.figshare.22117148.

## Box 1 Example of Randomized Response Technique (RRT) instructions provided to respondent when asking about sensitive topics. This design, which uses a randomizing process to determine how the respondent should answer, is known as a “forced-response” RRT.

Roll the die & answer the question using the following instructions:

- If you roll a **1, 2, 3,** or **4,** you must **answer** the question **honestly**
- If you roll a **5,** you must **answer yes,** regardless of whether this answer is true for you
- If you roll a **6,** you must **answer no,** regardless of whether this answer is true for you

Do not say which number you rolled

**In the last 12 months, have you entered a protected area to hunt wildlife?**
