# Supplementary material for: Topic sensitivity still affects honest responding, even when specialized questioning techniques are used
Source: Conserv Sci Pract. Author manuscript; Available in PMC 2023 Jun 27. (PMC7614702; doi:10.1111/csp2.12927)
Supplement: Supporting Information [file EMS177383-supplement-Supporting_Information.docx]

# Supplementary Materials

***Unpublished data***

Prior to conducting the surveys outlined in this manuscript, extensive preliminary research was conducted across the landscape (e.g., see Ibbett et al. *in review. A mixed method approach for measuring topic sensitivity in conservation*). Information identified in group exercises (but not reported in the manuscript) revealed information about the types of rule-breaking behaviours that occur in the landscape, as well as the characteristics of those who conduct them. These results were used to inform the study design.

Table S1a. Fields used to design the Open Data Kit form for the Main Study questionnaire.

| **label::English** | **label::Kiswahili** | **hint::English** | **hint::Kiswahili** |
| --- | --- | --- | --- |
| **Section 1: Location** | | | |
| Region | Region | For interviewer to answer | For interviewer to answer |
| District | District | For interviewer to answer | For interviewer to answer |
| What is the district? | What is the district? | For interviewer to answer | For interviewer to answer |
| Village | Village | For interviewer to answer | For interviewer to answer |
| Sub-village | Sub-village | For interviewer to answer | For interviewer to answer |
| Which is the nearest protected area? | Which is the nearest protected area? | For interviewer to answer | For interviewer to answer |
| Who is conducting the interview? | Who is conducting the interview? | For interviewer to answer | For interviewer to answer |
| What type of respondent is being interviewed? | What type of respondent is being interviewed? |  |  |
| Can you provide more information on why the respondent is part of the snowball sample? | Can you provide more information on why the respondent is part of the snowball sample? | "For interviewer to answer:  Were we given their information? Who gave us their information? What behaviours do we think they do? Did they approach us?" | "For interviewer to answer:  Were we given their information? Who gave us their information? What behaviours do we think they do? Did they approach us?" |
| What gender is the respondent? | 1. Jinsia ya mhojiwa? |  |  |
| **Section 2: Consent** | | | |
| "Read the information sheet to the respondent, either from the sheet of paper or the phone:  My name is ${interviewer} and I am a Research Assistant working for a project being conducted by Bangor University in the United Kingdom. Today, I would like to invite you to take part in a short study. The aim of the study is to understand what life is like for people like you that live close to a protected area. I am asking you to take part because you live close to a one. In total we are hoping to speak to between 2,000 and 3,000 people like you who live close to a protected area. If you agree to take part, I will ask you some questions and record your answer on this device [show questionnaire on tablet]. I will never record your name or your address on this, or any other form; your answers are private and confidential. The first set of questions is about you and your household, then I will ask about different activities you may or may not have done inside the protected area. Next, I will ask questions about your opinions and experiences of living in this area. This should take between 30 minutes and one hour. Because I am not recording your name, your answers cannot be attributed to you, and it will not be possible to identify you from our records. Information you provide will not be shared with anybody outside of the research team and will only be used by members of the research team based in the UK. Your answers, together with those of 2,000-3,000 other people, will be used to write reports. The study has been approved by the Bangor University Ethics Committee. If you would prefer not to take part, that is ok. Also, if you agree at first but then change your mind and would like to stop at any point, please tell me and I will stop immediately. If you have any concerns please contact us on this phone number [provide contact card]. Before going any further, do you have any questions that you would like to ask me? Would you like to take part in this study?" | "Read the information sheet to the respondent, either from the sheet of paper or the phone:  Jina langu naitwa ${interviewer} ni mtafiti msaidizi katika mradi unaotekelezwa na chuo kikuu cha Bangor kilichoko Uingereza. Ningependa kukukaribisha kushiriki katika utafiti huu mfupi. Lengo la utafiti huu ni kuelewa juu ya maisha yakoje kwa watu kama nyinyi mnaoishi karibu na hifadhi. Nakuomba kushiriki kwasababu unaishi eneo hili ambalo lipo karibu na [Jina la hifadhi]. Kwa ujumla tunatarajia kuzungumza na wanakijiji 2,000-3,000 ambao wanaishi karibu na hifadhi. Kama unakubali kushiriki nitakuuliza maswali machache na kujaza majibu yako kwenye kifaa hiki. Sitachukua jina lako au anwani yako na majibu yako yatakuwa ni siri. Kipengele cha kwanza cha maswali kinakuhusu wewe na kaya yako, halafu nitakuuliza kuhusu shughuli mbalimbali ambazo unawezakuwa umewahikufanya au hujawahi ndani ya hifadhi Baada ya hapo nitakuuliza maswali kuhusu maoni yako na uzoefu wako wa kuishi katika eneo hili. Mahojiano haya yatachukua muda wa dk45 mpaka saa moja. Kwasababu sitaandika jina lako Majibu yako hayatahusianishwa na wewe na haitawezekana kukutambua kutoka kwenye majibu tuliyoandika. Taarifa utakazozitoa hazitatolewa kwa mtu yoyote nje ya timu hii ya utafiti na itatumika tu na watafiti wa timu hii walioko Uingereza. Majibu yako pamoja na wale watu wengine 2,000-3,000 yatatumika kuandika ripoti. Utafiti huu umethibitishwa na Kamati ya Maadili ya Chuo kikuu cha Bangor. Kama hautapenda kushiriki katika mahojiano haya ni sawa, pia unaruhusiwa kuondoka kama utapata dharura au ukibadilisha mawazo wakati wowote tafadhali niambie na nitasitisha mahojiano mara moja. Je, una swali lolote la kuniuliza kabla hatujaendelea? Kama una wasiwasi wowote tafadhali wasiliana nasi kwa namba (mpe kadi ya mawasiliano) Je, utapenda kushiriki katika utafiti huu? " |  |  |
| "I confirm that I have had the ConHuB Information Sheet read out to me and I understand the information provided for this study. I have had the opportunity to ask questions if necessary and have had these answered satisfactorily. I understand that my participation is voluntary and that I am free to withdraw at any time without giving any reason. If I withdraw my data will be removed from the study and will be destroyed. I understand that the answers I provide will be used for the purposes detailed in the ConHuB Information Sheet. Based upon the above, I agree to take part in this study " | "Nakiri kwamba nimesoma/ kusomewa fomu ya [Conservation and Human Behavior-ConHuB] yaani Uhifadhi wa Mazingira na tabia za watu] na nimeelewa juu ya utafiti huu. Nilipewa nafasi ya kuuliza maswali na kupewa majibu yanayoridhisha. Naelewa kuwa kushiriki kwangu ni kwa hiari na niko huru kujitoa muda wowote nikipata dharura, na nikijitoa taarifa nilizotoa kwenye mjadala zitafutwa. Naelewa kwamba majibu niliyotoa yatatumika kwa kusudi lililotolewa kwenye fomu ya ConHuB ya ushiriki wa mahojiano Kwa taarifa hapo juu nakubali kushiriki kwenye mjadala huu " |  |  |
| Thank the respondent for their time | Thank the respondent for their time | If you want to return to the previous question, just swipe backwards. | If you want to return to the previous question, just swipe backwards. |
| **Section 3: COVID-19 Precautions** | | | |
| "While we understand that the government has announced that Tanzania has no COVID-19, because it is a dangerous disease, we are taking precautions to keep ourselves and everyone we meet safe in case there are cases that the government has not detected. " | Wakati tunaelewa kwamba Serikali ya Tanzania imetangaza kutokuwa na ugonjwa wa COVID-19, kwasababu ni ugonjwa wa hatari , tunachukua tahadhari ili kuhakikisha usalama wetu na wa kila mmoja tunayekutana naye endapo kuna maambukizi ambayo serikali haijagundua bado. | " If the guide has not already explained our COVID-19 precautions then explain to the respondent: 1. That the team are clear of symptoms 2. That we wear masks to protect ourselves and the respondent 3. That we will be working outside and maintaining social distancing 4. We will be washing hands frequently " | " If the guide has not already explained our COVID-19 precautions then explain to the respondent: 1. That the team are clear of symptoms 2. That we wear masks to protect ourselves and the respondent 3. That we will be working outside and maintaining social distancing 4. We will be washing hands frequently " |
| Does anyone in your household have symptoms of COVID-19 that have developed over the previous week? These are: a new and persistent cough, difficulty in breathing, a high fever, a recent loss of taste or smell? | Kuna mtu yeyote kwenye kaya yako ambaye ana dalili za COVID-19 ambazo zimeonekana wiki iliyopita? Hizi ni: kikohozi kipya na endelevu,kupumua kwa shida, homa kali, kupoteza uwezo wa kutambua harufu na ladha ya chakula hivi karibuni? |  |  |
| Thank the respondent for their time, explain that even though the sick individual may not have COVID-19 we do not want to put other respondents at risk if they do have COVID-19. Wish them or their household member a quick recovery | Thank the respondent for their time, explain that even though the sick individual may not have COVID-19 we do not want to put other respondents at risk if they do have COVID-19. Wish them or their household member a quick recovery |  |  |
| **Section 4: Respondent demographics** | | | |
| 1. I am going to ask you a few questions about yourself and the others in your household, by household I mean those who eat together and sleep in the same house, including children who may be at boarding school | Nitakuuliza mswali machache kuhusu wewe na watu wengine wa kaya yako, Ninaposema kaya ninamaanisha wale wote wanaokula pamoja na kulala kwenye nyumba moja ni kijuimuisha na watoto waliopo shule za bweni. |  |  |
| 2. How old are you? | 2. Una umri gani? | If the respondent does not know their age ask for a best estimate. To skip question enter -99 | If the respondent does not know their age ask for a best estimate. To skip question enter -99 |
| 3a. What tribe do you belong to? | 3a. Wewe ni kabila gani? |  |  |
| 3b. Please specify which tribe | 3b. Tafadhali taja kabila lako |  |  |
| 4. How many years have you lived in this village? | 4. Umeishi kwenye kijiji hiki kwa miaka mingapi? | Round to the nearest whole number (e.g. 5 months = 0 yrs, 6 months = 1 yr). To skip question enter -99 | Round to the nearest whole number (e.g. 5 months = 0 yrs, 6 months = 1 yr). To skip question enter -99 |
| 5. Are you currently attending school? | 5. Je, unasoma shule kwa sasa? |  |  |
| 6. How many years of schooling have you completed? | 6. Umesoma shule kwa miaka mingapi? | Includes primary and secondary school. To skip question enter -99 | Includes primary and secondary school. To skip question enter -99 |
| 7. How many other people live in this household? Both adults and children | 7. Watu wangapi wengine unaishi nao kwenye kaya yako? Watu wazima na watoto |  |  |
| **Section 5a: RRT questions for men** | | | |
| Introducing RRT | Introducing RRT | "I’m now going to ask you some questions in a different way. The way that I will ask you these questions is a bit like a game, and like games, there are rules that you must follow.  First of all I will give you a dice and this cup, and ask you to roll the dice inside this cup, like this [shake dice], giving it a really good shake.  I will then ask you to look inside the cup to see what number the dice is showing [look inside cup].  If you roll a **1, 2, 3 or 4** you should answer my question **with the truth about your actual behaviour,** your answer can be **Yes** or **No.**  If you roll a **5** you must answer my question **Yes** *Even if this is not the true answer*  If you roll a **6** you must answer my question **No** *Even if this is not the true answer*  It is important that you do not tell me what number your dice shows, that is your secret.  Let’s have a practice, first, I will answer a question.  See, I’m shaking the dice in the cup, now I’m going to look inside & see what number I got. OK. So I know what number I have and now I will look at this sheet to remind myself how to answer: 1, 2, 3, or 4 and I must answer according to my own behaviour, my answer can be Yes or No. If I got a 5, I just say Yes and if I got a 6, I just say No.  So, now let’s think of a question… Did I break the speed limit in that car when I was in town last week? I’m looking at my dice number again [look in cup], OK, my answer is Yes. Now, you see, you do not know if I said Yes because I really did drive too fast last week, or if I said Yes because the dice landed on 5. This means that you do not know anything sensitive about me, we are both safe.  Let’s have a practice. This time, you roll the dice in the cup. Make sure you give it a really big shake and then look inside the cup to see what number you got.  My question is: Have you eaten beans today?  Following the rules of the dice, what is your answer, remember if your dice landed on 1, 2, 3, or 4 you must answer according to your own behaviour, your answer can be Yes or No. However, if you get a 5, you just say Yes and if you got a 6, you just say No [point to dice card]. OK, so your answer was X [say answer given], but see, I can’t tell if you answered X [say answer given], because it was your true behaviour, or if it is because your dice landed on X [say 5 if answer was Yes, say No if answer was 6].  Do you understand?  **Repeat example until respondent understands how to answer** OK, I can see that you now understand this method. I will now ask you the actual questions. " | "Sasa nitakuuliza baadhi ya maswali kwa njia tofauti. Namna nitakavyokuuliza haya maswali ni kama mchezo, na kama tunavyojua kwa kila mchezo kuna kanuni ambazo unapaswa kufuata.  Kwanza kabisa nitakupa kete na hiki kikombe, na nitakuomba utikise kete ndani ya hiki kikombe, kama hivi [Tikisa kete], tikisa kete sawasawa kabisa.  Nitakuomba utazame ndani ya kikombe kuona namba gani imetokea kwenye kete[Tazama ndani ya kikombe].  Kama ukitikisa kete ikatokea **1,2,3 au 4** unapaswa kujibu swali langu [kulingana na shughuli unazofanya] jibu lako linaweza kuwa **NDIYO** au **HAPANA**.  Kama ukitikisa kete ukapata **5** ni lazima ujibu **NDIYO** kwenye swali langu. * Hata Kama hili sio jibu sahihi*  Kama ukitikisa kete ukapata **6** ni lazima ujibu **HAPANA** kwenye swali langu * Hata Kama hili sio jibu sahihi*  Ni muhimu kutokuniambia ni namba gani umepata kwenye kete yako, hiyo ni siri yako.  Sasa tufanye jaribio kwanza, nitajibu swali. Tazama, natikisa kete ndani ya kikombe, na sasa nitatazama ndani ya kikombe kuona ni namba ngapi ya kete nimepata. Sawa. Kwa hiyo sasa najua ni namba ngapi nimepata, na sasa nitatazama kwenye kadi hii kujikumbusha namna ya kujibu: 1,2,3, au 4 ninapaswa kujibu kulingana na shughuli ninazofanya. Kama nimepata 5,nitasema tu NDIYO na kama nimepata namba 6, nitasema tu HAPANA.  Sasa tufikirie kuhusu swali…. Je, nilizidisha kiwango cha mwendo nilipokuwa nikiendesha gari mjini wiki iliyopita? Ninaangalia namba niliyopata kwenye kete yangu tena {Angalia ndani ya kikombe}. Sawa, jibu langu ni NDIYO Sasa, unaona,huwezi kujua kama nimesema NDIYO kwasababu kweli nilizidisha mwendo wa gari wiki iliyopita, au nimesema NDIYO kwasbabu kete imeonyesha namba 5. Hii inamaanisha kwamba haufahamu chochote nyeti kuhusu mimi, kwahiyo wote tuko salama. Sasa tujaribu.  Wakati huu zungusha kete ndani ya kikombe. Hakikisha unatikisa sawasawa kisha angalia ndani ya kikombe kuona umepata namba ngapi.  Swali langu ni kwamba: Je, umekula maharage leo?  Kwa kufuata kanuni ya kete, jibu lako ni nini, kumbuka kama kete imeonyesha namba 1,2,3 au 4 unapaswa kujibu kulingana na shughuli unazofanya, jibu lako linaweza kuwa NDIYO au HAPANA. Ingawa ukipata namba 5, unapaswa kujibu NDIYO na kama ukipata 6, unapaswa kujibu HAPANA (Onyesha kwenye kadi ya kete). SAWA. Hivyo jibu lako lilikuwa X{Taja jibu lililotolewa},lakini ona, siwezi kusema kama umejibu X {Taja jibu lililotolewa}, kwasababu ndio shughuli yako ya kweli unayoifanya, au kwasababu kete imeonyesha namba X{sema 5 kama jibu lilikuwa NDIO, sema HAPANA kama jibu lilikuwa 6}.  Je, umeelewa?  **Rudia mfano mpaka muhojiwa atakapoelewa namna ya kujibu** Sasa naona umeelewa mbinu hii, hivyo nitakuuliza maswali halisi. " |
| 1. How many times did you practice the method before the respondent understood? | 1. How many times did you practice the method before the respondent understood? | For interviewer to answer. To skip question enter -99 | For interviewer to answer. To skip question enter -99 |
| 2. In the past 12 months have you hunted wildlife inside ${protected_area_name} for example birds francolin, guinea fowl, quail, small animals like dik dik or impala, or larger animals like buffalo, giraffe or others? | 2. Kwa miezi 12 iliyopita uliwinda ndani ya ${protected_area_name} kwa mf Ndege: Kware, Kanga, wanyama wadogo: Digi digi, swala, wanyama wakubwa: Nyati, Twiga au wengine? |  |  |
| 3. In the past 12 months have you been fishing inside ${protected_area_name}? | 3. Kwa miezi 12 iliyopita umekuwa ukivua samaki ndani ya ${protected_area_name}? |  |  |
| 4. In the past 12 months have you taken livestock inside ${protected_area_name} to graze or for water? | 4. Kwa miezi 12 iliyopita ulipeleka mifugo ndani ya ${protected_area_name} kwaajili ya malisho au maji? |  |  |
| 5. In the past 12 months have you collected timber or construction materials inside ${protected_area_name}? | 5. Kwa miezi 12 iliyopita ulivuna mbao au vifaa vya kujengea ndani ya ${protected_area_name}? |  |  |
| 6. In the past 12 months have you entered ${protected_area_name} without a permit? | 6. Kwa miezi 12 iliyopita uliingia ndani ya ${protected_area_name} bila kibali? |  |  |
| **Section 5b: RRT questions for women** | | | |
| Introducing RRT | Introducing RRT | "I’m now going to ask you some questions in a different way. The way that I will ask you these questions is a bit like a game, and like games, there are rules that you must follow.  First of all I will give you a dice and this cup, and ask you to roll the dice inside this cup, like this [shake dice], giving it a really good shake.  I will then ask you to look inside the cup to see what number the dice is showing [look inside cup].  If you roll a **1, 2, 3 or 4** you should answer my question **with the truth about your actual behaviour,** your answer can be **Yes** or **No.**  If you roll a **5** you must answer my question **Yes** *Even if this is not the true answer*  If you roll a **6** you must answer my question **No** *Even if this is not the true answer*  It is important that you do not tell me what number your dice shows, that is your secret.  Let’s have a practice, first, I will answer a question.  See, I’m shaking the dice in the cup, now I’m going to look inside & see what number I got. OK. So I know what number I have and now I will look at this sheet to remind myself how to answer: 1, 2, 3, or 4 and I must answer according to my own behaviour, my answer can be Yes or No. If I got a 5, I just say Yes and if I got a 6, I just say No.  So, now let’s think of a question… Did I break the speed limit in that car when I was in town last week? I’m looking at my dice number again [look in cup], OK, my answer is Yes. Now, you see, you do not know if I said Yes because I really did drive too fast last week, or if I said Yes because the dice landed on 5. This means that you do not know anything sensitive about me, we are both safe.  Let’s have a practice. This time, you roll the dice in the cup. Make sure you give it a really big shake and then look inside the cup to see what number you got.  My question is: Have you eaten beans today?  Following the rules of the dice, what is your answer, remember if your dice landed on 1, 2, 3, or 4 you must answer according to your own behaviour, your answer can be Yes or No. However, if you get a 5, you just say Yes and if you got a 6, you just say No [point to dice card]. OK, so your answer was X [say answer given], but see, I can’t tell if you answered X [say answer given], because it was your true behaviour, or if it is because your dice landed on X [say 5 if answer was Yes, say No if answer was 6].  Do you understand?  **Repeat example until respondent understands how to answer** OK, I can see that you now understand this method. I will now ask you the actual questions. " | "Sasa nitakuuliza baadhi ya maswali kwa njia tofauti. Namna nitakavyokuuliza haya maswali ni kama mchezo, na kama tunavyojua kwa kila mchezo kuna kanuni ambazo unapaswa kufuata.  Kwanza kabisa nitakupa kete na hiki kikombe, na nitakuomba utikise kete ndani ya hiki kikombe, kama hivi [Tikisa kete], tikisa kete sawasawa kabisa.  Nitakuomba utazame ndani ya kikombe kuona namba gani imetokea kwenye kete[Tazama ndani ya kikombe].  Kama ukitikisa kete ikatokea **1,2,3 au 4** unapaswa kujibu swali langu [kulingana na shughuli unazofanya] jibu lako linaweza kuwa **NDIYO** au **HAPANA**.  Kama ukitikisa kete ukapata **5** ni lazima ujibu **NDIYO** kwenye swali langu. * Hata Kama hili sio jibu sahihi*  Kama ukitikisa kete ukapata **6** ni lazima ujibu **HAPANA** kwenye swali langu * Hata Kama hili sio jibu sahihi*  Ni muhimu kutokuniambia ni namba gani umepata kwenye kete yako, hiyo ni siri yako.  Sasa tufanye jaribio kwanza, nitajibu swali. Tazama, natikisa kete ndani ya kikombe, na sasa nitatazama ndani ya kikombe kuona ni namba ngapi ya kete nimepata. Sawa. Kwa hiyo sasa najua ni namba ngapi nimepata, na sasa nitatazama kwenye kadi hii kujikumbusha namna ya kujibu: 1,2,3, au 4 ninapaswa kujibu kulingana na shughuli ninazofanya. Kama nimepata 5,nitasema tu NDIYO na kama nimepata namba 6, nitasema tu HAPANA.  Sasa tufikirie kuhusu swali…. Je, nilizidisha kiwango cha mwendo nilipokuwa nikiendesha gari mjini wiki iliyopita? Ninaangalia namba niliyopata kwenye kete yangu tena {Angalia ndani ya kikombe}. Sawa, jibu langu ni NDIYO Sasa, unaona,huwezi kujua kama nimesema NDIYO kwasababu kweli nilizidisha mwendo wa gari wiki iliyopita, au nimesema NDIYO kwasbabu kete imeonyesha namba 5. Hii inamaanisha kwamba haufahamu chochote nyeti kuhusu mimi, kwahiyo wote tuko salama. Sasa tujaribu.  Wakati huu zungusha kete ndani ya kikombe. Hakikisha unatikisa sawasawa kisha angalia ndani ya kikombe kuona umepata namba ngapi.  Swali langu ni kwamba: Je, umekula maharage leo?  Kwa kufuata kanuni ya kete, jibu lako ni nini, kumbuka kama kete imeonyesha namba 1,2,3 au 4 unapaswa kujibu kulingana na shughuli unazofanya, jibu lako linaweza kuwa NDIYO au HAPANA. Ingawa ukipata namba 5, unapaswa kujibu NDIYO na kama ukipata 6, unapaswa kujibu HAPANA (Onyesha kwenye kadi ya kete). SAWA. Hivyo jibu lako lilikuwa X{Taja jibu lililotolewa},lakini ona, siwezi kusema kama umejibu X {Taja jibu lililotolewa}, kwasababu ndio shughuli yako ya kweli unayoifanya, au kwasababu kete imeonyesha namba X{sema 5 kama jibu lilikuwa NDIO, sema HAPANA kama jibu lilikuwa 6}.  Je, umeelewa?  **Rudia mfano mpaka muhojiwa atakapoelewa namna ya kujibu** Sasa naona umeelewa mbinu hii, hivyo nitakuuliza maswali halisi. " |
| 1. How many times did you practice the method before the respondent understood? | 1. How many times did you practice the method before the respondent understood? | For interviewer to answer. To skip question enter -99 | For interviewer to answer. To skip question enter -99 |
| 2. In the past 12 months have you encouraged someone in your household to hunt wildlife inside ${protected_area_name} for example birds francolin, guinea fowl, quail, small animals like dik dik or impala, or larger animals like buffalo, giraffe or others? | 2. Kwa miezi 12 iliyopita ulimshauri mtu yoyote kwenye kaya yako kuwinda wanyamapori ndani ya ${protected_area_name} mf Ndege: Kware au Kanga Wanyama wadogo: Digidigi au Swala, Wanyama wakubwa: Nyati, Twiga au wengine? |  |  |
| 3. In the past 12 months have you encouraged someone in your household to fish inside ${protected_area_name}? | 3. Kwa miezi 12 iliyopita ulimshauri mtu yoyote kwenye kaya yako kuvua samaki ndani ya ${protected_area_name}? |  |  |
| 4. In the past 12 months have you encouraged someone in your household to take livestock inside ${protected_area_name} to graze or for water? | 4. Kwa miezi 12 iliyopita ulimshauri mtu yoyote kwenye kaya yako kupeleka mifugo ndani ya ${protected_area_name} kwaajili ya malisho au maji? |  |  |
| 5. In the past 12 months have you encouraged someone in your household to collect timber or construction materials inside ${protected_area_name}? | 5. Kwa miezi 12 iliyopita ulimshauri mtu yoyote kwenye kaya yako kuvuna mbao au vifaa vya kujengea ndani ya ${protected_area_name}? |  |  |
| 6. In the past 12 months have you entered ${protected_area_name} without a permit? | 6. Kwa miezi 12 iliyopita uliingia ndani ya ${protected_area_name} bila kibali? |  |  |
| **Section 6: Review of RRT** | | | |
| 7. ${interviewer}, how well do you think the respondent understood the method? | 7. ${interviewer}, unafikiri mhojiwa alielewa mbinu hii vizuri kiasi gani? |  |  |
| 8. ${interviewer}, any comments on RRT section? | 8. ${interviewer}, any comments on RRT section? | For interviewer, if no comments leave blank | For interviewer, if no comments leave blank |
| **Section 7: Thank you** |  |  |  |
| We are interested in learning about how people’s lives change over time. Would you be willing to be visited again by our team in approximately 12 months time?  If so I will record your name and some contact details, therefore your responses will no longer be anonymous. However, your name and phone number will be stored securely, and will not be shared with anybody outside the research team. | Tunapenda kujifunza jinsi maisha ya watu yanavyobadilika kadiri muda unavyokwenda. Je utakuwa tayari kushiriki mahojiano yajayo na timu yetu katika kipindi cha miezi kumi na mbili ijayo?  Kama Ndio, nitaandika jina lako, namba ya simu na jira nukta ya nyumba yako ili tuweze kukupata tutakapo rudi. Licha ya kupata taarifa hizi majibu yako yataendelea kuwa siri na hayatatolewa kwa mtu yoyote nje ya timu ya utafiti wala kuonyesha utambulisho wako popote |  |  |
| 2. What is your name so we can find you again in 12 months time? | 2. Tafadhali niambie jina lako ili tuweze kukutafuta tena baada ya muda wa miezi 12 |  |  |
| 3. What is your common name? | 3. Jina lako maarufu ni lipi? |  |  |
| 4. What is your phone number so we can find you again in 12 months time? | 4. Tafadhali nitajie namba yako ya simu ili tuweze kukutafuta tena baada ya muda wa miezi 12 |  |  |
| 5. GPS location | 5. GPS location | If you are not at the respondent's house then SKIP this question. Automatically records the GPS location when accuracy is less than 20m. You may have to be outside to get a good location | If you are not at the respondent's house then SKIP this question. Automatically records the GPS location when accuracy is less than 20m. You may have to be outside to get a good location |
| Thank the respondent for their time | Mshukuru mhojiwa kwa muda wake |  |  |
| ${interviewer}. where is the interview being conducted? | ${interviewer}. where is the interview being conducted? | For interviewer to answer | For interviewer to answer |
| ${interviewer}, what is the "other" location? | ${interviewer}, what is the "other" location? | For interviewer to answer | For interviewer to answer |
| ${interviewer}, any notes to record? Or unusual circumstances? E.g. other people present, disruptions etc | ${interviewer}, any notes to record? Or unusual circumstances? E.g. other people present, disruptions etc | For interviewer to answer | For interviewer to answer |
| End of questionnaire | | | |

Table S1b. Fields used to build the experimental study questionnaire in Open Data Kit.

| **label::English** | **label::Kiswahili** | **hint::English** | **hint::Kiswahili** |
| --- | --- | --- | --- |
| **Section 1: Location** | | | |
| Who is conducting the interview? | Nani anafanya mahojianao? |  |  |
| Region | Mkoa |  |  |
| District | Wilaya |  |  |
| Name of the village | Jina la kijiji | Please write the village name. E.g. Iringa | Andika jina la kijiji. |
| Name of sub-village | Jina la kitongoji | Write the name of the sub-village | Andika jina la kitongoji |
| Nearest Protected Area | Hifadhi iliyokaribu hapa ni ipi | **Do not ask the respondent this** | **Usimwulize mhojiwa hili** |
| **Section 2: Consent** | | | |
| Read the Consent Script to the participant | Soma fomu ya kuomba ridhaa ya mhojiwa | Hello. My name is ${interviewer} and I am helping X, who is from X University in the UK to conduct research. X’s research is all about understanding the best way to ask questions about natural resource use.  We are asking lots of people to complete this survey so that we can understand how people prefer to answer questions about natural resource use. The information you provide us is very important in making sure that we ask questions in the right way.   The questionnaire will take about 25 minutes to complete. Any information you provide will be anonymous, this means I will not record your name, or any information that can personally identify you or your household. Your answers will not be communicated to anyone in a form where your reply can be linked to you. I will record all your answers on this phone. All your answers will then be saved on a secure computer which can only be accessed by XXX.   At the end of the research, XXX will write a report on her findings. This report will be used to help other researchers conduct research that better meets the needs of local people. Some results may also be published internationally so that other people in different countries can learn from our experience working with communities here.  Please note that we are independent, we are not related to the government or any NGOs and we have neutral views. We have permission of the Tanzanian government and the village chief to carry out this research. However, participation is voluntary. You do not have to participant and you stop participating at any time, without explanation. If you do I will discard your responses. If you feel uncomfortable answering some of the questions, you do not have to answer. If you would like to skip a question or a topic, please say.  This study has been reviewed by, and received ethics clearance through X University. If you have any questions, please ask me and I will do my best to answer them.   If you remain unhappy or wish to make a formal complaint, I can give you the contact details of someone to discuss this with. | Habari, Jina langu ni ${interviewer} na ninamsaidia X anayetoka chuo kikuu cha X Uingereza, kufanya utafiti. Utafiti wa X unahusu kuelewa njia nzuri ya kuuliza maswali kuhusu utumiaji wa rasilimali.   Tutauliza watu wengi kujibu maswali ya utafiti huu ili tuweze kuelewa jinsi watu wanavyopenda kujibu maswali kuhusu matumizi ya rasilimali asili. Taarifa utakayotupatia ni ya muhimu sana katika kuhakikisha kwamba tutauliza maswali kwakutumia njia sahihi.  Mahojiano yanakadiriwa kuchukua takribani dakika 25. Taarifa yoyote utakayotupatia itakuwa ya siri, hii inamaana sitaandika jina lako au taarifa yoyote ambayo itaweza kukutambulisha binafsi au kaya yako. Majibu yako hayatatolewa kwa mtu yoyote kwa njia yoyote ambayo mtu anaweza kuhusianisha majibu yako na wewe. Nitaandika majibu yako yote kwenye simu hii. Majibu yako yote yatahifadhiwa kwenye kompyuta salama ambayo inaweza kutumiwa na XXX tu.  Mwisho wa utafiti XXX ataandika ripoti ya majibu ya utafiti. Taarifa hii itatumika kusaidia watafiti wengine kufanya tafiti zinazokidhi mahitaji ya jamii. Matokeo mengine yanaweza kuchapishwa kimataifa ili watu wengine katika mataifa mbalimbali waweze kujifunza kupitia uzoefu wetu wa kufanya kazi katika jamii hizi.  Tafadhali kumbuka tunajitegemea na hatuhusiani na upande wowote, uwe wa Serikali au Mashirika yasiyokuwa ya serikali na tunamtazamo usiofungamana na upande wowote. Tuna kibali kutoka Serikali ya Tanzania na Serikali ya kijiji ya kufanya utafiti huu. Japokuwa ushiriki ni wa hiari. Unaweza kuwa huru kujiondoa wakati wowote bila kutoa sababu. Kama utajiondoa nitafuta majibu yako. Kama hautakuwa huru kujibu baadhi ya maswali unarususiwa kutokujibu. Kama utataka kuruka swali au mada tafadhali sema.  Utafiti huu umerejewa na kupewa kibali cha kimaadili kupitia chuo kikuu cha X. Kama una swali lolote, tafadhali niulize na nitajitahidi niwezavyo kukujibu.  Kama utakuwa na wasiwasi au unataka kutoa malalamiko nitakupa mawasiliano ya mtu unayeweza kuzungumza naye. |
| Did participant give their consent to participate? | Je, mhojiwa ametoa ridhaa yake ya ushiriki? |  |  |
| Thank the participant and end the survey. | Mshukuru mshiriki na maliza mahojiano. |  |  |
| What was the gender of the participant? | Taja jinsia ya mshiriki |  |  |
| **Section 3: Covid-19 Precautions** | | | |
| While we understand that the government has announced that Tanzania has no COVID-19, because it is a dangerous disease, we are taking precautions to keep ourselves and everyone we meet safe in case there are cases that the government has not detected. | Wakati tunaelewa kwamba Serikali ya Tanzania imetangaza kutokuwa na ugonjwa wa COVID 19, kwasababu ni ugonjwa wa hatari, tunachukua tahadhari ili kuhakikisha usalama wetu na wa kila mmoja tunayekutana naye endapo kuna maambukizi ambayo serikali haijagundua bado. | If the guide has not already explained our COVID precautions then explain to the respondent:  1. That the team are clear of symptoms  2. That we wear masks to protect ourselves and the respondent  3. That we will be working outside and maintaining social distancing  4. We will be washing hands frequently | If the guide has not already explained our COVID precautions then explain to the respondent:  1. That the team are clear of symptoms  2. That we wear masks to protect ourselves and the respondent  3. That we will be working outside and maintaining social distancing  4. We will be washing hands frequently |
| Does anyone in your household have symptoms of COVID19 that have developed over the previous week? | Kuna mtu yeyote kwenye kaya yako ambaye ana dalili za COVID19 ambazo zimeonekana wiki iliyopita? | These are:  • a new and persistent cough • difficulty in breathing • a high fever, • a recent loss of taste or smell | Hizi ni:  • kikohozi kipya na endelevu • kupumua kwa shida • homa kali • kupoteza uwezo wa kutambua harufu na ladha ya chakula hivi karibuni |
| Thank the respondent for their time, explain that even though the sick individual may not have COVID we do not want to put other respondents at risk if they do have COVID. Wish them or their household member a quick recovery | Thank the respondent for their time, explain that even though the sick individual may not have COVID we do not want to put other respondents at risk if they do have COVID. Wish them or their household member a quick recovery |  |  |
| Section 4:  Participant demographics | | | |
| What gender is the participant? | Jinsia ya mshiriki |  |  |
| How old are you? | Una umri gani? | If unknown, ask them to estimate their age | Kama hawafahamu waombe wakadirie umri wao |
| What tribe are you? | Wewe ni kabila gani? |  |  |
| Please specify which tribe | Tafadhari ainisha kabila lako |  |  |
| What is the main language you speak? | Ipi ni lugha yako ya msingi unayozungumza? |  |  |
| Please specify which language | Tafadhali taja lugha hiyo |  |  |
| How many years of schooling do you have? | Umesoma kwa miaka mingapi? | Enter approximate number of years   If none, enter 0  If unsure, write DK For diploma/degree - add on the number of extra years e.g. 13 + 2 = 15 | Weka kadirio la miaka    Kama hakuna weka 0  Kama hana uhakika, andika DK Kwa Stashahada/Shahada- ongeza idadi ya miaka zaidi |
| Can you read? | Unaweza kusoma? |  |  |
| How do you find reading? | Ni rahisi kiasi gani kwako kusoma? | Read out options to respondent | Soma machaguo kwa mhojiwa |
| **Section 5: Introducing RRT** | | | |
| Intro: Testing Methods | Utangulizi: Kujaribu mbinu | The aim of this next section is to find out how you most prefer to answer questions.   When we are asked questions about using natural resources, sometimes we don’t always want to tell the truth.  We might be embarrassed about our answer, we might not trust the person asking the questions, or we might be scared that if we do tell the truth we will get into trouble.   However, for researchers, when we ask questions about natural resource it is very important that we do get honest answers.   Otherwise the information we have will not be correct, and we may not make the best recommendations for the community. | Lengo la kipengele kifuatacho ni kufahamu ni jinsi gani ungependa kujibu maswali.  Tunapoulizwa maswali juu ya matumizi ya rasilimali asili, wakati mwingine hatupendi kusema ukweli.  Tunaweza tukaona aibu juu ya majibu yetu, tunaweza tusimwamini mtu anayetuuliza maswali au tunaweza tukaogopa kwamba tutaingia matatizoni tukisema ukweli.  Japokuwa kwetu sisi watafiti tunapouliza maswali kuhusu rasilimali asili ni muhimu sana kupata majibu sahihi na ya kweli.  La sivyo taarifa tutakayoipata haitakuwa sahihi na hatutaweza kutoa mapendekezo mazuri kwa jamii. |
| Introduce method | Kutambulisha mbinu | To solve this problem, researchers have developed special ways of asking questions, which allow people to answer questions honestly, but mean the researcher cannot tell if the person answering the question does the activity.   I would like to try one of these ways with you, to see if you understand it and your opinions.   I do not want to know whether this is something you or your household does.   I am only interested in understanding the best way for a researcher to ask questions about this. | Ili kutatua tatizo hili,watafiti wamegundua njia malumu ya kuuliza maswali ambayo inawaruhusu watu kutoa majibu ya kweli,lakini mtafiti hawezi kusema kama mhojiwa anajihusisha na shughuli hizo.  Ningependa kujaribu moja ya mbinu hizi na wewe ili kuona kama unaielewa na kupata maoni yako.  Sihitaji kufahamu kama ni kitu ambacho wewe au kaya yako mnakifanya, ninapenda tu kuelewa njia sahihi kwa mtafiti kuuliza maswali kuhusu hili. |
| Introduce the characters | Tambulisha Mhusika | To make sure that you do not answer questions about yourself I would like to introduce you to these fictional characters.  Here I have 14 characters. Each of whom owns or does different things.   When we try the different ways of answering questions, I want you to pretend you are one of these characters, and so you should give me the answer that they should give.   Do you understand? | Ili kuhakikisha haujibu maswali haya kuhusu wewe mwenyewe ningependa kukufahamisha kuhusu watu wa kubuni.  Hapa nina watu 14 (wakubuni). Kila mmoja anamiliki au kufanya shughuli mbalimbali.   Tunapojaribu mbinu mbalimbali za kujibu maswali, ningependa ufikirie wewe ni mmoja wa watu hawa wa kubuni na unipe majibu ambayo wangenipa.   Je, umeelewa? |
| Collect character card for Moja | Chukua kadi ya Mtu Namba Moja | Collect character card for Moja  For example, here we have Moja. Moja eats several different types of fruit. These are: • Mango • Avocado • Papaya • Pineapple | Kwa mfano, hapa tunaye Mtu Namba Moja. Mtu Namba Moja anakula matunda ya aina tofauti, nayo ni: • Embe • Parachichi • Papai • Nanasi |
| **Section 6: Practicing RRT** | | | |
| **Dice method** | **Mbinu ya Kete** | The way to answer this question is a bit like a game. And like games, there are rules you must follow.  First of all I will give you a dice, and ask you to roll it.   If you roll a **1, 2, 3 or 4** you should answer my question truthfully  If you roll a **5** you must always say **Yes** *Even if this is not the true answer*  If you roll a **6** you must always say **No** *Even if this is not the true answer*  Do you understand?  Let’s have a practice. *[Roll the dice]* which number did you get? What answer should you give?  Let’s practice with an example.   Here is Moja. My question to Moja is “Moja, do you eat avocado?”   I roll the dice. I get XX. Because I got a XX, my answer should be XX.   Do you understand? | Jinsi ya kujibu maswali haya ni kama mchezo na katika kila mchezo kuna sheria ambazo unapaswa kuzifuata.  Kwanza kabisa nitakupa kete uizungushe.  Kama ukizungusha kete na ukapata **1, 2, 3, au 4** unapswa **kusema kweli**  Kama ukizungusha kete na ukapata **5** lazima useme **Ndiyo** *Hata kama ndiyo sio jibu sahihi, bado unapaswa kusema ndiyo*  Kama ukizungusha kete na ukapata **6** unapaswa kusema **Hapana** *Hata kama hapana sio jibu la kweli unapaswa kusema hapana*  Unaelewa?  Sasa tujaribu *[Zungusha kete]* Umepata namba gani? Ni Jibu gani unapswa kutoa?  Tujaribu kwa mfano.  Huyu ni Mtu Namba Moja. Swali langu kwa “Mtu Namba Moja, je, unakula parachichi?”  Nazungusha kete. Nimepata XX, jibu langu linapswa kuwa XX  Umeelewa? |
| Was the respondent familiar with a dice? | Mhojiwa alikuwa anafahamu kuhusu kete? | Yes / No / Prefer not to answer | Ndiyo / Hapana / Napenda kutojibu |
| *Select character card for Mbili* | *Chagua kadi kwa Mtu Namba Mbili* | Ok, now we shall practice with Mbili. Mbili eats: Mango Watermelon Orange Banana | Sasa tutajaribu na Mtu Namba Mbili.  Mtu Namba Mbili unakula: Embe Tikiti Chungwa Ndizi |
| Dice method  Mbili, do you eat avocado? | Kanuni ya Kete  Mtu Namba Mbili, je, unakula parachichi? | Remember, if you roll a: 1, 2, 3, 4 **answer truthfully** 5 say **YES** 6 say **NO**  Record the number of practices required, before the respondent answered correctly.   If respondent Prefers not to answer enter '999' | Kumbuka, kama ukirusha kete ukapata: **1, 2, 3 au 4** jibu swali kwa **ukweli** **5** sema **ndiyo** **6** sema **hapana**  Andika idadi ya majaribio yaliyofanyika kabla ya muhojiwa kutoa jibu sahihi  Kama mhojiwa hapendi kujibu andika '999' |
| **Section 7: Collecting data – This section was repeated 6 times, once for each of the study behaviours** | | | |
| Select the card for **${character 1}** | Chagua kadi kwa Mtu Namba **${character 1}** | Briefly describe the activities on the card.  ${character 1_attributes} | Elezea kwa kifupi shughuli zilizoandikwa kwenye kadi:  ${character 1_attributes} |
| Dice method  ${character 1}, do you ${behaviour 1}? | Mbinu ya Kete   Mtu Namba ${character 1}, ${behaviour 1}? | Refer to the 'protected area' as the ${pa_type}.   Remember, if you roll a: 1, 2, 3, or 4 answer honestly 5 say Yes 6 say No | Rejea ‘Hifadhi’ kama ${pa_type}.  Kumbuka, kama ukirusha kete ukapata: **1, 2, 3 au 4** jibu swali kwa **ukweli** **5** sema **ndiyo** **6** sema **hapana** |
| From the answer you gave, do you think I would be able to tell if ${character 1}, ${behaviour 1}? | Kutokana na jibu ulilotoa, unafikiri nitaweza kusema kama Mtu Namba ${character 1}, ${behaviour 1}? |  |  |
| What number did you roll on the dice? | Umepata upande wa kete wenye namba ngapi? | So I can check if you answered correctly.   If NA, enter '0' | Ili niangalie kama majibu yako ni sahihi  Kama mhojiwa hapendi kujibu andika 0 |
| Select the card for **${character 2}** | Chagua kadi kwa Mtu Namba **${character 2}** | Briefly describe the activities on the card.  ${character 2_attributes} | Elezea kwa kifupi shughuli zilizoandikwa kwenye kadi:  ${character 2_attributes} |
| Dice method  ${character 2}, do you do behaviour 1? | Mbinu ya Kete   Mtu Namba ${character 2}, ${behaviour 1}? | Refer to the 'protected area' as the ${pa_type}.  Remember, if you roll a: 1, 2, 3, or 4 answer honestly 5 say Yes 6 say No | Rejea ‘Hifadhi’ kama ${pa_type}. Kumbuka, kama ukirusha kete ukapata: **1, 2, 3 au 4** jibu swali kwa **ukweli** **5** sema **ndiyo** **6** sema **hapana** |
| From the answer you gave, do you think I would be able to tell if ${character 2} ${behaviour 1}? | Kutokana na jibu ulilotoa, unafikiri nitaweza kusema kama Mtu Namba ${character 2}, ${behaviour 1}? |  |  |
| What number did you roll on the dice? | Umepata upande wa kete wenye namba ngapi? | So I can check if you answered correctly.  If NA, enter '0' | Ili niangalie kama majibu yako ni sahihi Kama mhojiwa hapendi kujibu andika 0 |
| **Section: 8 Review of RRT** | | | |
| Do you feel you clearly understood how to answer the questions? | Unafikiri umeelewa vizuri namna ya kujibu maswali? |  |  |
| How easy did you find it to answer the question using this method? | Ni kwa kiasi gani umeona ni rahisi kujibu maswali kwa kutumia mbinu hii? |  |  |
| How secret do you think your answers were using this method? | Unafikiri majibu yako ni ya usiri kiasi gani kwa kutumia mbinu hii? |  |  |
| How comfortable would you feel answering questions honestly about sensitive topics using this method? | Je, utakuwa huru kiasi gani kujibu maswali kwa ukweli kuhusu mada nyeti kwa kutumia mbinu hii? |  |  |
| Any other comments to add? | Una maoni yoyote ya kuongezea? | Record any comments from the respondent about the method | Kitu cho chote wanachosema kuhusu mbinu hii |
| **Section 9: Interviewer feedback** | | | |
| The survey is now finished. Thank you for participating. | Mwisho wa mahojiano Asante kwa kushiriki | Do you have any questions for me? | Una maswali yoyote ya kuniuliza? |
| ${interviewer}, how well do you think the respondent understood the method? | ${interviewer}, unafikiri mhojiwa alielewa mbinu hii vizuri kiasi gani? |  |  |
| ${interviewer}, did you feel the respondent was deliberately answering incorrectly? | ${interviewer}, ulihisi kama mhojiwa alikuwa anakusudia kukosea majibu? | E.g. they were scared to answer honestly | Mfano, alikuwa anaogopa kutoa majibu ya kweli |
| ${interviewer}, how engaged was the participant throughout the survey? | ${interviewer}, je muhojiwa ameonyesha ushirikiano kwa kiwango gani? |  |  |
| ${interviewer}, how did you find surveying this individual? | ${interviewer}, kumhoji huyu mtu kulikuwaje? |  |  |
| Have you any comments or feedback? | Una maoni yoyote au mrejesho? | Record any comments or feedback If none, NA | Andika maoni yoyote au mrejesho |


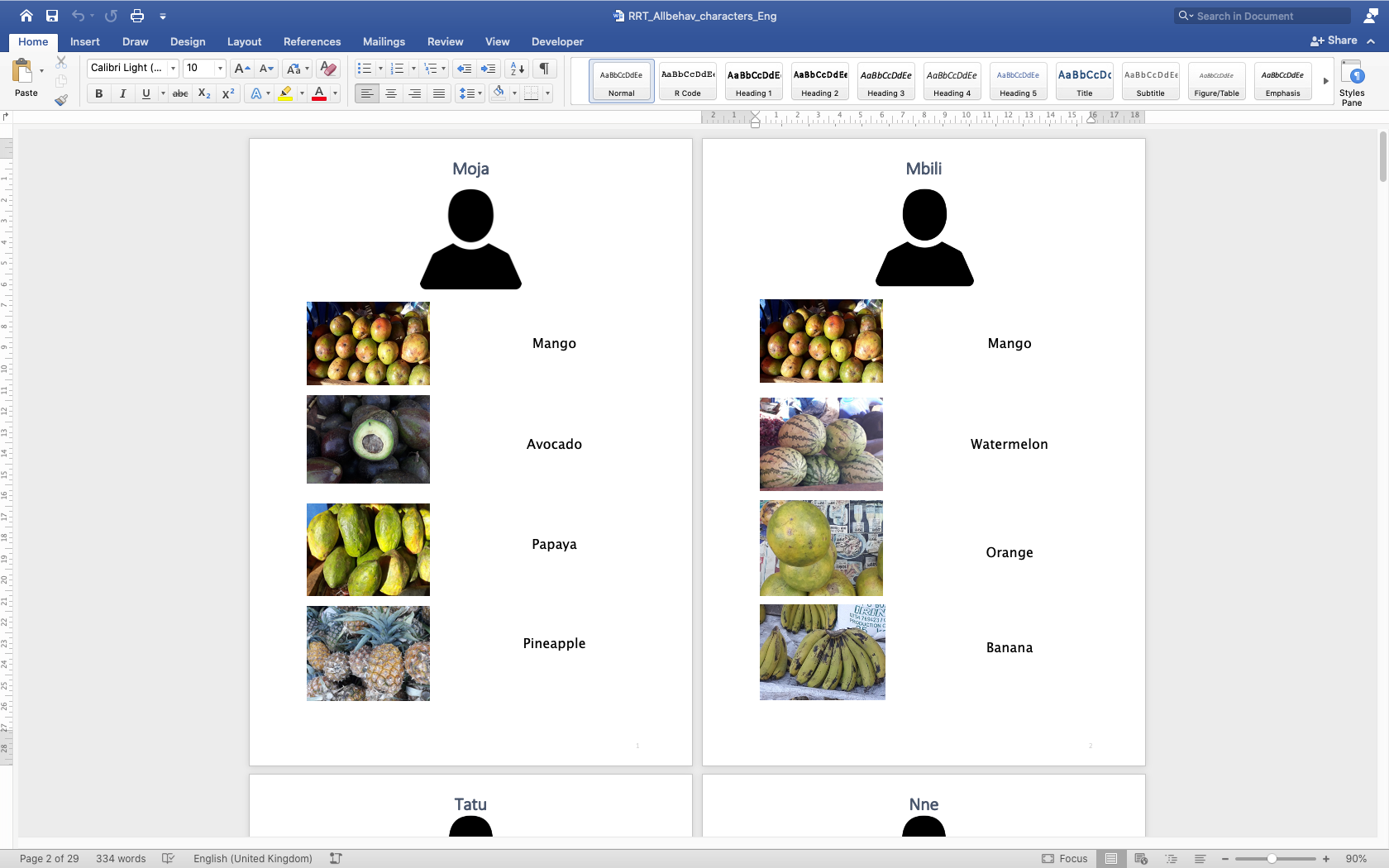


Figure S1a. Examples of character cards used to ask respondents about the behaviour of fictional characters. In total, 14 different cards were shown to respondents, in a random order. The cards show here were used for the practice exercise, which was about consuming fruit.

Figure S1b. Plotted regression coefficients with standard errors from a general linear mixed model from the experimental study of whether a respondent answered the Randomised Response Technique question correctly or not, with random effects for respondent. *Reference levels: Female, Required to answer no to sensitive behaviour.


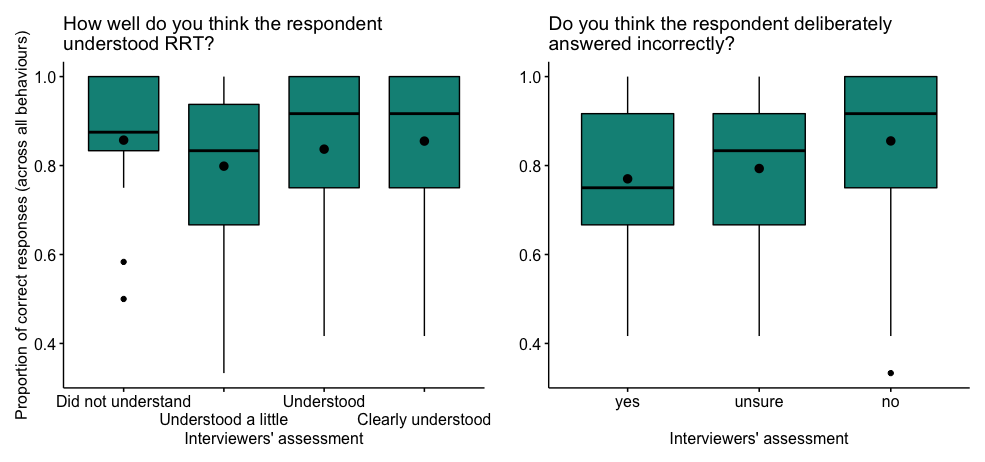


Figure S1c. Interviewers’ assessments of respondents understanding and evasive responses, compared against respondents’ performance (measured by the proportion of correct responses given, across all behaviours). Dots show the mean, thick lines in shaded boxes show the median.

Table S1c. Feedback from respondents about the RRT method, coded into three different categories.

| **Comment (translated to English)** | **Positive** | **Concerns** | **Understanding** |
| --- | --- | --- | --- |
| He was afraid, as he thought it [RRT] contained magic | - | 1 | - |
| He was afraid of [rolling the] number five. He asked why he should have to answer he did, when he did not? | - | 1 | - |
| He says this method has a bit of secrecy | - | - | - |
| Fearful people will not answer questions using this [RRT] method. | - | 1 | - |
| He said some of the respondents will not answer 'yes' for rule breaking because of sensitivity. | - | 1 | - |
| He was worried his farm is going to be included in the Protected area as a result of this study. Consequently, he deliberately answered incorrectly. | - | 1 | - |
| I am afraid that I may be asked to give evidence of rule breaking after participating in this survey | - | 1 | - |
| I think it will be difficult especially for people who did not go to school | - | - | 1 |
| I was fearful of giving wrong answers on the method | - | 1 | - |
| Improve the method by avoiding repetition of questions and pictures on the cards | - | - | - |
| Is a good method | 1 | - | - |
| Is a good method for it gives freedom | 1 | - | - |
| It is difficult for us who did not go to school | - | - | 1 |
| It is difficult to know what these things mean | - | - | 1 |
| It is simple and easy to understand | 1 | - | - |
| It needs much energy to understand, and it will be more difficult to understand to those who did not go to school | - | - | 1 |
| It was difficult at the beginning but as I progressed it became easy | 1 | - | - |
| It was difficult because I didn't get prior information [that surveys were taking place], so I didn’t get prepared to participate in the survey. | - | - | 1 |
| It will not work for people who did not go to school | - | - | 1 |
| It’s a good method | 1 | - | - |
| It’s difficult at the beginning but as I progressed it became clear. | 1 | - | - |
| It’s easy | 1 | - | - |
| It’s had to answer ‘yes’ to rule breaking, but I am concerned the study may bring eviction. | - | 1 | - |
| It will confuse people especially those who have not studied [been to school] | - | - | 1 |
| This technique is good | 1 | - | - |
| The method is good, but it forces one to give answers even if it is not true | - | 1 | - |
| Hard if you do not read, it is disturbing | - | - | 1 |
| It is difficult because it contains many things that are confusing | - | - | 1 |
| It's a good technique | 1 | - | - |
| It's easy | 1 | - | - |
| Nice method and would yield success | 1 | - | - |
| The dice method is easy to answer questions | 1 | - | - |
| People are now educated, so you are better [off] asking them directly | - | - | 1 |
| People with difficulties to understand will face problems, because it is confusing | - | - | 1 |
| She associated dice with witchcraft and refused to respond to the questions | - | 1 | - |
| She associated RRT with witchcraft | - | 1 | - |
| The method seems to be good and [it] is my hope you will get good answers | 1 | - | - |
| This method is difficult | - | - | 1 |
| This method is easy but for people like the Sukuma it will be more difficult because they are so worried | 1 | 1 | - |
| Understanding of this method depends on understanding the capacity of a respondent. | - | - | 1 |
| [The respondent] understood the method at the beginning but later forgot due to many numbers of character cards. | - | - | 1 |
| **Total number of comments** | **14 (31%)** | **12 (30%)** | **14 (31%)** |
